# Outcomes of a Heart Failure Telemonitoring Program Implemented as the Standard of Care in an Outpatient Heart Function Clinic: Pretest-Posttest Pragmatic Study

**DOI:** 10.2196/16538

**Published:** 2020-02-06

**Authors:** Patrick Ware, Heather J Ross, Joseph A Cafazzo, Chris Boodoo, Mikayla Munnery, Emily Seto

**Affiliations:** 1 Centre for Global eHealth Innovation Techna Institute University Health Network Toronto, ON Canada; 2 Ted Rogers Centre for Heart Research University Health Network Toronto, ON Canada; 3 Department of Medicine University of Toronto Toronto, ON Canada; 4 Peter Munk Cardiac Centre University Health Network Toronto, ON Canada; 5 Institute of Health Policy, Management and Evaluation Dalla Lana School of Public Health University of Toronto Toronto, ON Canada; 6 Institute of Biomaterials and Biomedical Engineering University of Toronto Toronto, ON Canada

**Keywords:** telemonitoring, telemedicine, virtual care, mHealth, heart failure

## Abstract

**Background:**

Telemonitoring (TM) can improve heart failure (HF) outcomes by facilitating patient self-care and clinical decisions. The *Medly* program enables patients to use a mobile phone to record daily HF readings and receive personalized self-care messages generated by a clinically validated algorithm. The TM system also generates alerts, which are immediately acted upon by the patients’ existing care team. This program has been operating for 3 years as part of the standard of care in an outpatient heart function clinic in Toronto, Canada.

**Objective:**

This study aimed to evaluate the 6-month impact of this TM program on health service utilization, clinical outcomes, quality of life (QoL), and patient self-care.

**Methods:**

This pragmatic quality improvement study employed a pretest-posttest design to compare 6-month outcome measures with those at program enrollment. The primary outcome was the number of HF-related hospitalizations. Secondary outcomes included all-cause hospitalizations, emergency department visits (HF related and all cause), length of stay (HF related and all cause), and visits to the outpatient clinic. Clinical outcomes included bloodwork (B-type natriuretic peptide [BNP], creatinine, and sodium), left ventricular ejection fraction, and predicted survival score using the Seattle Heart Failure Model. QoL was measured using the Minnesota Living with Heart Failure Questionnaire (MLHFQ) and the 5-level EuroQol 5-dimensional questionnaire. Self-care was measured using the Self-Care of Heart Failure Index (SCHFI). The difference in outcome scores was analyzed using negative binomial distribution and Poisson regressions for the health service utilization outcomes and linear regressions for all other outcomes to control for key demographic and clinical variables.

**Results:**

Available data for 315 patients enrolled in the TM program between August 2016 and January 2019 were analyzed. A 50% decrease in HF-related hospitalizations (incidence rate ratio [IRR]=0.50; *P*<.001) and a 24% decrease in the number of all-cause hospitalizations (IRR=0.76; *P*=.02) were found when comparing the number of events 6 months after program enrollment with the number of events 6 months before enrollment. With regard to clinical outcomes at 6 months, a 59% decrease in BNP values was found after adjusting for control variables. Moreover, 6-month MLHFQ total scores were 9.8 points lower than baseline scores (*P*<.001), representing a clinically meaningful improvement in HF-related QoL. Similarly, the MLHFQ physical and emotional subscales showed a decrease of 5.4 points (*P*<.001) and 1.5 points (*P*=.04), respectively. Finally, patient self-care after 6 months improved as demonstrated by a 7.8-point (*P*<.001) and 8.5-point (*P*=.01) increase in the SCHFI maintenance and management scores, respectively. No significant changes were observed in the remaining secondary outcomes.

**Conclusions:**

This study suggests that an HF TM program, which provides patients with self-care support and active monitoring by their existing care team, can reduce health service utilization and improve clinical, QoL, and patient self-care outcomes.

## Introduction

### Background

Heart failure (HF) is estimated to affect more than 1 million Canadians [1] and 6.5 million adults in the United States [2], many of whom experience chronic symptoms of fatigue and shortness of breath, punctuated by sporadic episodes of decompensation [3]. The unpredictability of these episodes leads to more HF hospitalizations compared with other conditions, representing a significant burden on health systems [4]. For patients with HF, hospitalizations and daily symptoms have a negative impact on daily functioning and ultimately their quality of life (QoL) [5].

Existing medical interventions, including pharmaceutical treatments, have been successful in prolonging the lives of patients with HF. However, with the exception of heart transplantation, full recovery is unlikely. Similar to many other chronic conditions, guideline-directed medical therapy also calls for patients with HF to play an active role through self-management of their diet, fluid restriction, and adherence to the medication schedules [6]. Although many patients with HF receive education for HF self-management during face-to-face clinic visits with care providers [6], mechanisms to support self-care between planned visits are needed to support patients once they go back to living their daily lives.

Telemonitoring (TM), which uses noninvasive electronic devices to collect and transmit physiological and disease-related data collected in patients’ homes to a care provider, can provide this self-care support [5,7], particularly when the TM system includes an algorithm that can provide targeted personalized feedback [8]. When combined with timely data transmission to clinicians, which can enable the early detection and remote clinical intervention of symptom exacerbations [9], TM has the potential to optimize HF management. This is supported by several meta-analyses of randomized controlled trials (RCTs), which have concluded that TM reduces the risk of mortality and the number of hospitalizations when compared with the standard of care [10-13]. Most recently, results from the large Telemedical Interventional Management in Heart Failure II study found that HF TM significantly reduced the percentage of days lost because of unplanned cardiovascular hospital admissions and all-cause death [14]. However, this evidence generally comes from efficacy trials, which are designed to measure an intervention’s impact under ideal conditions [15]. Such conditions are attained through the use of restrictive inclusion criteria and additional resources (eg, implementation staff, training plans, and trial supervision) aimed at ensuring intervention uptake and appropriate use [16]. In fact, the results of high-profile neutral HF TM trials [17-19] have been largely attributed to problems in the intervention’s delivery and uptake [20]. As real-world interventions tend to have broader inclusion criteria and more barriers to appropriate use, it is not uncommon for them to demonstrate less benefit compared with the outcomes of similar interventions in more controlled trials [15].

The overarching trend toward positive evidence in efficacy trials is likely sufficient to encourage many health organizations to make HF TM available to their patient populations. However, questions remain about when, how, and under what conditions HF TM interventions should be delivered. Such questions are best answered by what has been termed practice-based evidence [16], which is the output of research that emphasizes an understanding of context through the use of pragmatic and mixed method study designs often seen in the quality improvement evaluations of real-world health services [21].

### Study Objectives

The objective of this study was to evaluate the 6-month impact of an HF TM program, called *Medly*, on health service utilization, clinical outcomes, QoL, and patient self-care. A published protocol outlined the mixed method quality improvement evaluation of the implementation and impact of the *Medly* program [22], which is implemented as part of the standard of care in an outpatient heart function clinic in Toronto, Canada.

## Methods

### Study Design

This pragmatic quality improvement study employed a pretest-posttest design to compare impact outcome measures after 6 months of enrollment with those at baseline. The study period spanned from August 23, 2016 (the date when the first patient was enrolled), to June 31, 2019, and was conducted at an ambulatory heart function clinic at the Peter Munk Cardiac Centre (PMCC). The PMCC is a part of the University Health Network (UHN), which is a large university-affiliated organization composed of 5 hospitals and institutes located in Toronto. The UHN Research Ethics Board (16-5789) approved the study as a quality improvement project. Under this definition, data generated as part of the standard of care could be analyzed for quality improvement purposes. However, collecting additional research data through questionnaires required informed consent from patients. Therefore, although all patients entering the program were invited to consent to complete patient-reported outcome questionnaires, this consent was not required to analyze the health service utilization and laboratory data that were retrospectively collected as part of this study.

### The Intervention

The *Medly* program features a clinically validated algorithm [23] to provide patients with personalized self-care messages and to alert members of their core HF care team when clinical intervention may be required. By outsourcing much of the self-care support to the algorithm, clinician resources are freed to manage more urgent cases within minutes of receiving patient data. This, according to the US Food and Drug Administration, is a form of active monitoring [24]. In contrast, passive TM is when patient data get transmitted but a clinician is not expected to take immediate clinical action [24], as is the case if a TM system cannot contextualize data based on urgency or if the telehealth clinician does not have rapid access to the patient’s most responsible physician (MRP) to make a necessary change to the patient’s care plan. The *Medly* program is hypothesized to improve patient self-care and enable early clinical intervention at the onset of symptom exacerbations. This, in turn, is expected to reduce avoidable health service utilization and improve HF clinical outcomes as well as patients’ QoL.

#### Telemonitoring System

**Figure 1:**
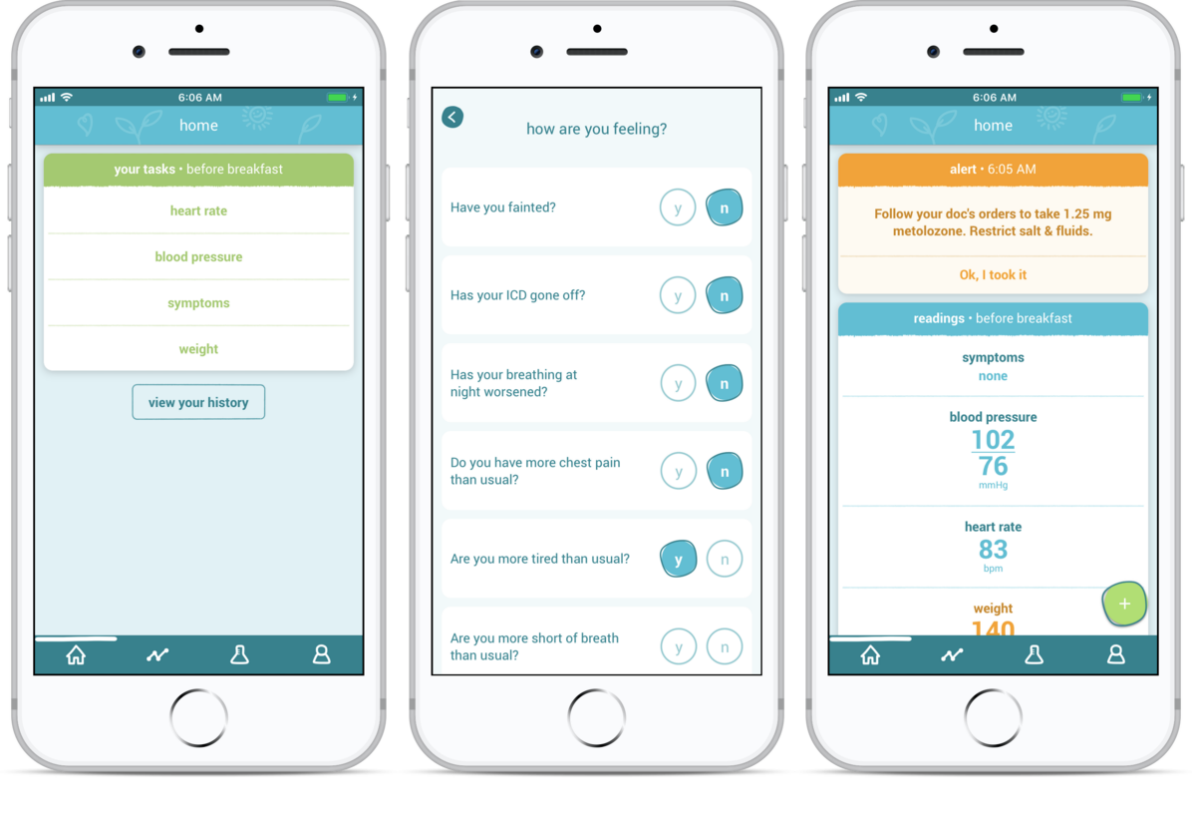
Pages of the *Medly* app showing the incomplete morning card with required readings, the symptoms questionnaire, and personalized self-care feedback after all 4 readings were taken and processed by the algorithm.

The *Medly* system includes a patient-facing app, which can be downloaded into an iOS or Android smartphone. The app enables patients to record weight, blood pressure, and heart rate using peripheral weight scales and blood pressure monitors. These data can be transmitted automatically to the *Medly* app via Bluetooth or entered manually. In addition, patients manually report symptoms by answering *yes* or *no* to a short series of questions (as seen in Figure 1). Once entered, these measures are processed by the algorithm embedded within the app that classifies a patient’s current health status into 1 of 9 states based on whether a value (or a clinically meaningful combination of multiple values) is above or below target thresholds, which have been set by the clinical team. The states (ie, algorithm outputs) determine which self-care messages are displayed to patients within the app. Examples of the self-care feedback messages include confirming with patients when everything is normal, instructing patients to take their prescribed diuretic medication when the change in weight is above the set threshold (ie, evidence of fluid retention), and suggesting when to contact their care providers or go to the emergency department (ED). The details of the algorithm’s development and its clinical validation have been published [23]. Other features of the *Medly* app include the ability to view graphical trends of each reading’s values and, to assist with adherence, an automated phone call to remind patients if they have not yet taken morning readings by 10 AM.

**Figure 2:**
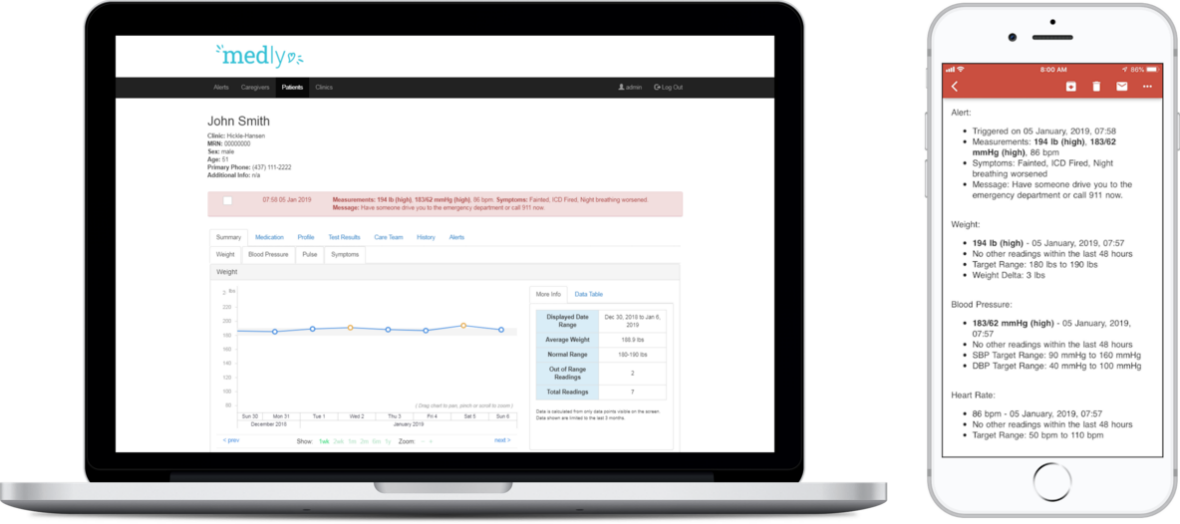
Patient profile in the Web-based *Medly* clinician dashboard and an example of an email alert message.

The algorithm also triggers alerts destined to clinical members of the patients’ care team, which can be delivered via email or viewed in the Web-based *Medly* dashboard, which currently stands apart from the hospital electronic medical record (EMR). Email alerts are contextualized by indicating which parameter or parameters triggered the alert, which is presented alongside the patient’s current medication list, latest HF-related laboratory results, and patient contact information. Similarly, this contextual information is also available on the *Medly* dashboard in addition to longitudinal graphs of each parameter measured and laboratory results. As such, the *Medly* dashboard is primarily used to actively manage periods of HF instability, but it can also be used when the patient is stable (eg, during follow-up visits), as it provides a holistic and longitudinal snapshot of the patient’s health. The *Medly* system was developed at the UHN, and all data collected resides in secure UHN servers. An example of the patient profile in the *Medly* dashboard has been illustrated in Figure 2

#### Intended Use: Supporting Clinical and Operational Services

The *Medly* program is intended to complement and not replace existing services. As such, the treating cardiologist presents the *Medly* program to a patient as a therapeutic option, and a decision regarding enrollment is made jointly between both parties. After a patient agrees to enter the program, they meet with a technical support staff member to begin the onboarding process, which includes an assessment of the patient’s equipment needs. Patients who require all pieces of equipment are provided with a *Medly* kit that includes a smartphone, which has been paired with an A&D Bluetooth–enabled weight scale and blood pressure cuff. For patients using their own smartphone, the technical support staff helps them download the *Medly* app from the Apple or Google Play store. If patients are missing one or both peripheral devices, they can borrow the missing device from the clinic for the duration of enrollment. Rationale and details of the bring your own device (BYOD) model have been published [25]. After setting up the equipment, the staff member then trains the patient on how to use the system and sets the target thresholds (based on the MRP’s instructions) to customize the algorithm. The entire onboarding process (ie, account creation, training, study consent, and equipment management) takes approximately 30 min. If technical issues are experienced, patients are instructed to contact the technical support staff member who helps them troubleshoot the problem and replace the equipment if necessary.

Unlike many other HF TM programs and trials, the *Medly* program does not have a defined end date. Rather, patients can stay in the program indefinitely or until there is no longer a clinical need (eg, patient receives a heart transplant). Regardless of duration, patients are expected to take their readings every day, first thing in the morning. The clinical response to TM alerts follows a triage structure during business hours with a frontline clinician (typically a registered nurse [RN] or nurse practitioner [NP] embedded within the care team in the outpatient clinic) who reviews alerts in the *Medly* dashboard and coordinates with the wider circle of care. Assuming a caseload of roughly 300 patients, a single frontline coordinator will typically receive and manage between 45 and 60 alerts per day. If required, more serious alerts or issues outside the frontline clinician’s scope of practice are escalated to the MRP. When adapting this program to fit clinic workflows, the MRPs opted to receive all email alerts so that when issues were escalated, they could easily retrieve all the relevant information from their email without having to log in to the dashboard. To ensure 7-day per week coverage, MRPs know that there is no frontline clinician working; therefore, it is up to them to manage all the alerts received in their email on weekends. Previous studies on the implementation of the program confirm that the intervention was being used by patients and clinicians as intended and that satisfaction was high among patients and clinicians [26,27].

#### Adaptable Components of the Medly Program

A qualitative study identified program components that can be adapted to ensure sustainability and fit within a site’s existing workflows, culture, and resources while maintaining the key ingredients needed to deliver the program’s intended outcomes [25]. First, the types of peripheral devices used are adaptable (ie, it does not matter if patients use their own device or borrow standardized equipment nor does it matter if data are transferred automatically via Bluetooth). Second, the professional qualifications of the frontline clinical staff members are adaptable, provided they have some experience in cardiology. These findings informed moving toward a BYOD model for those who already have the necessary equipment. Another change is that when the program started, the frontline clinical and technical support roles were played by NPs and a telehealth analyst, respectively. However, since May 2018, both roles are being performed by a single RN who is still embedded within the outpatient clinic but who actively monitors all patients enrolled in the *Medly* program.

### Study Participants

Participants in this study included all those who were enrolled in the *Medly* program between August 23, 2016, and January 31, 2019 (6 months before the end of the study period). To be eligible for the program, patients had to meet the following criteria: (1) aged 18 years or older, (2) diagnosed with HF and followed by a cardiologist at the heart function clinic, (3) can speak and read English (or have an informal caregiver who does), and (4) are able to comply with using *Medly*. In addition, clinicians use clinical judgment in determining whether they believe a patient will benefit. Considerations typically include disease severity (eg, New York Heart Association [NYHA] classification class 2 or 3), a need for self-care support, and a perception that patients will be engaged enough to take daily readings.

### Outcome Measures

Outcomes to evaluate the pre-post impact of the *Medly* program over a 6-month period are classified into 4 categories: (1) health service utilization, (2) clinical outcomes, (3) QoL, and (4) self-care.

#### Health Service Utilization

The primary outcome was the number of HF-related hospitalizations. Secondary health service utilization outcomes included the number of all-cause hospitalizations, number of visits to the ED (HF related and all cause), length of stay (HF related and all cause), and number of visits to the outpatient clinic. Baseline values represented a count of events occurring 6 months before enrollment to the date of enrollment. Follow-up values represented a count of events occurring from the date of enrollment to the calendar date, 6 months following enrollment. Finally, the length of stay was defined as the cumulative number of days spent as an inpatient over the periods defined above.

#### Clinical Outcomes

HF-related clinical outcomes primarily included laboratory tests routinely done as part of HF management, including B-Type Natriuretic Peptide (BNP), which is secreted by the heart in response to stretch from pressure or volume overload [28]. As such, BNP is a key HF prognostic indicator, with higher levels being associated with an increased risk of mortality and hospitalization. Additional clinical outcomes included creatinine, sodium, and left ventricular ejection fraction (LVEF). Finally, given the natural decline of HF, we also sought to measure the impact of the *Medly* program on predicted survival via the Seattle Heart Failure Model (SHFM) score [29].

#### Quality of Life

HF-specific QoL was assessed using the Minnesota Living with Heart Failure Questionnaire (MLHFQ), which is composed of 21 items, in which participants rate their perceptions of the degree to which HF and its treatment impacts their daily life on a 6-point Likert scale ranging from 0 (meaning no impairment) to 5 (meaning very much impaired). Therefore, lower scores indicate better HF-specific QoL, and an increase or decrease in 5 points is considered the minimal clinically significant change [30]. The MLHFQ yields a total QoL score and a score for the physical and emotional well-being subscales. In addition, the 5-level EuroQol 5-dimensional (EQ-5D-5L) questionnaire was used as a measure of generic health status [31], with total EQ-5D-5L scores being calculated based on a time trade-off value set derived for Canada by Xie et al [32].

#### Self-Care

The Self-Care of Heart Failure Index (SCHFI) was used to measure changes in patients’ self-care [33]. Unlike the MLHFQ, the SCHFI does not produce a total score but rather a standardized score between 0 and 100 for the scales of *maintenance* (behaviors aimed at maintaining physiologic stability), *management* (response to symptoms when they occur), and self-care *confidence*. A score above 70 on each subscale is considered adequate, and an 8-point difference is considered to be the minimally important change [34].

### Demographic and Control Variables

Demographic and clinical characteristics were collected to describe the study population, and a subset of these was used as control variables in the impact analyses to increase the likelihood that any observed changes in the outcomes could be attributed to the *Medly* program. Selected control variables included sex, age at enrollment, NYHA class, LVEF at enrollment (categorized as reduced ejection fraction [LVEF <40%] vs preserved ejection fraction [≥40%]), location of enrollment (inpatient ward vs outpatient clinic), and duration followed at the outpatient clinic (<6 months vs >6 months). The latter is based on results from a previous RCT evaluating the *Medly* system, which found that new patients (regardless of treatment arm) improved more than long-term patients because of the confounding effect of being enrolled at the outpatient clinic [35]. 

### Data Collection

Data for the health service utilization outcomes, clinical outcomes, inputs for the SHFM, and available demographic and control variables were extracted from patients’ EMRs and the *Medly* program’s administrative records. Laboratory values at baseline and 6 months were taken from laboratory tests performed closest to the actual baseline or 6-month date within a 2-month window.

Questionnaires for the remaining demographic information and the QoL and self-care outcomes were administered to patients who consented. Baseline questionnaires were given to patients during the enrollment session, and although patients were encouraged to complete it before leaving, they were permitted to take it home and return the completed questionnaire using prepaid postage. The 6-month questionnaires were mailed to patients at the appropriate time with instructions to complete and return it using prepaid postage.

### Data Analysis

Although many patients are enrolled in the *Medly* program indefinitely, the intended primary analysis was to compare baseline outcome values with those at 6 months [22]. Paired-sample *t* tests and Wilcoxon signed-rank tests were originally planned [22]; however, we ultimately opted to perform multivariate regressions to allow for the controlling of possible confounders.

Linear regressions (ordinary least squares method) of the aforementioned control variables were performed to analyze differences in the QoL, self-care, and clinical outcomes. These regressions required the transformation of non-normal outcome data when applicable (ie, cubic transformation for EQ-5D-5L data and log transformation for the BNP and creatinine data). Finally, the Breusch-Pagan test was used to test the presence of heteroscedasticity [36]. If found, the linear regressions were reported with robust standard errors to correct for heteroscedasticity [37], as was the case for the BNP and sodium linear regressions.

Most of the health service utilization outcomes were regressed with negative binomial distribution to account for the presence of overdispersion [38]. An exception was the analysis for HF-related hospitalizations, which used Poisson regression because no overdispersion was detected. As, by definition, patients new to the outpatient clinic would have a lower number of visits at their baseline measure relative to the number of visits at 6 months, an interaction term between time and duration followed at the outpatient clinic was added to this regression. All outcome data generated by patients who entered the program during the study period were analyzed under the intention-to-treat principle. However, because health service utilization data could not be generated following a person’s death, patients who died before completing 6 months in the program were excluded from the health utilization analyses to avoid biasing the results in a positive direction.

Baseline and 6-month descriptive statistics for each outcome variable (before adjusting for the control variables) and descriptive statistics for the variables used to characterize the study population were obtained using SPSS version 24 (IBM Corporation). The data transformations and regressions were conducted in RStudio version 1.0.153 (RStudio Inc). All statistical tests results were 2-tailed, and a *P* value of less than .05 (*P*<.05) was used to indicate statistical significance.

## Results

### Characteristics of Study Participants

A total of 315 patients were enrolled in the program during the study period, of which 255 consented to complete questionnaires (211 patients returned a baseline questionnaire and 156 returned a completed 6-month questionnaire).

Participants of the *Medly* program were predominantly men (245/315, 77.8%), with an average age of 58.3 years (SD 15.5). With regard to clinical characteristics, approximately half experienced relatively mild daily HF symptoms with 47.1% (143/304) of patients being classified as NYHA class 2 or less at the time of program enrollment, and the average LVEF of patients was 31.8% (SD 13.4). Three-fourth (235/315, 74.6%) of the participants were enrolled during regularly scheduled outpatient visits, whereas 25.5% (80/315) were enrolled from the inpatient ward before being sent home following a hospital stay. Slightly more than half (183/315, 58.1%) had been followed by the HF clinic for more than 6 months at the time of enrollment, with the remaining being considered new to the clinic. Additional patient characteristics are presented in Multimedia Appendix 1.

Of the 315 patients who entered the program during the analysis period, 30 patients were no longer enrolled after 6 months: 57% (17/30) were removed for clinical reasons (eg, received a heart transplant, recovered, and became palliative), 27% (8/30) left for personal reasons (eg, perception that the benefits were not worth the effort and life circumstances), and 17% (5/30) of these patients died. A comprehensive analysis of why patients were removed or chose to leave the *Medly* program was published elsewhere [27].

### Impact of the Medly Program

The descriptive statistics for the baseline and 6-month outcome values before adjusting for the control variables are presented in Table 1.

**Table 1:** Descriptive statistics for baseline and 6-month outcome variables.

Outcomes			Baseline				6 months		
			N		Mean (SD)		N		Mean (SD)
**Health service utilization**									
	Hospitalizations (HF^a^ related)	309		0.46 (0.71)		309		0.23 (0.51)	
	Hospitalizations (all cause)	308		0.64 (0.89)		308		0.49 (0.97)	
	Length of stay (HF related)	309		5.9 (11.1)		309		4.5 (14.6)	
	Length of stay (all cause)	308		7.4 (12.4)		308		6.2 (17.1)	
	ED^b^ visits (HF related)	309		0.04 (0.21)		309		0.02 (0.14)	
	ED visits (all cause)	308		0.13 (0.48)		308		0.17 (0.54)	
	Outpatient clinic	308		1.9 (1.8)		308		2.7 (2.2)	
**Clinical outcomes**									
	B-type natriuretic peptide (pg/mL)	277		701.4 (757.5)		216		540.3 (725.2)	
	Sodium (mmol/L)	282		137.7 (3.1)		223		137.9 (3.0)	
	Creatinine (µmol/L)	282		123.9 (52.1)		223		131.6 (59.7)	
	Left ventricular ejection fraction (%)	308		32.1 (13.6)		274		33.4 (13.3)	
	Seattle Heart Failure Model	315		0.85 (0.94)		315		0.82 (0.94)	
**Quality of life**									
	MLHFQ^c^—total	211		53.2 (26.3)		156		42.4 (26.0)	
	MLHFQ—physical	211		22.9 (11.8)		156		17.4 (11.9)	
	MLHFQ—emotional	211		12.0 (7.5)		156		10.2 (7.6)	
	5-level EuroQol 5-dimensional questionnaire	208		0.79 (0.12)		153		0.81 (0.12)	
**Self-care**									
	SCHFI^d^—maintenance	210		70.9 (16.8)		156		78.5 (13.9)	
	SCHFI—management	142		64.2 (21.9)		66		72.5 (19.1)	
	SCHFI—confidence	209		67.2 (20.4)		154		69.7 (20.2)	

^a^HF: heart failure.

^b^ED: emergency department.

^c^MLHFQ: Minnesota Living with Heart Failure Questionnaire.

^d^SCHFI: Self-Care of Heart Failure Index.

#### Health Service Utilization

For the primary outcome, the number of HF-related hospitalizations decreased from a mean (minimum to maximum, SD) of 0.46 (0-4, 0.71) to 0.23 (0-3, 0.51). After adjusting for the control variables, the Poisson regression found a statistically significant incidence rate ratio (IRR) of 0.50 (*P*<.001), comparing the number of HF-related hospitalizations between 6 month and baseline (Table 2). This can be interpreted as a 50% reduction in the number of HF-related hospitalizations.

**Table 2:** Poisson and negative binomial regressions showing the effect of 6 months in the *Medly* program on the number of heart failure–related and all-cause hospitalizations when controlled for key demographic and clinical variables.

Variables	Heart failure–related hospitalizations Poisson regression^a^			All-cause hospitalizations negative binomial regression^b^			
	Coefficient (SE)	IRR^c^ (SE)	*P* value		Coefficient (SE)	IRR	*P* value
6-month follow-up	−0.69 (0.15)	0.50 (0.07)	<.001		−0.28 (0.12)	0.76 (0.09)	.02
Onboarded from ward	1.21 (0.15)	3.36 (0.52)	<.001		1.27 (0.13)	3.55 (0.45)	<.001
Left ventricular ejection fraction <40%	0.00 (0.16)	1.00 (0.15)	.98		−0.18 (0.14)	0.84 (0.84)	.20
New York Heart Association class	0.15 (0.06)	1.16 (0.07)	.013		0.18 (0.05)	1.20 (1.20)	<.001
Age (years)	0.00 (0.00)	1.00 (0.004)	.68		0.003 (0.004)	1.00 (0.004)	.54
Female	0.01 (0.18)	1.01 (0.17)	.95		−0.002 (0.16)	1.00 (0.16)	.99
New to outpatient clinic	−0.02 (0.15)	0.98 (0.15)	.90		−0.08 (0.13)	0.92 (0.12)	.52
Intercept	−1.53 (0.38)	N/A^d^	<.001		−1.41 (0.34)	N/A	<.001

^a^Number of observations = 606.

^b^Number of observations = 604.

^c^IRR: incidence rate ratio.

^d^N/A: not applicable.

The number of all-cause hospitalizations also decreased from an average of 0.64 (0-7, 0.89) to 0.49 (0-6, 0.97) after 6 months. The results of the negative binomial regression, also shown in Table 2, confirm that this represents a significant reduction in all-cause hospitalizations of 24% (IRR=0.76; *P*=.02). Regressions for length of stay (HF related and all cause), ED visits (HF related and all cause), and outpatient clinic visits found no significant difference between baseline and 6 months as shown in Multimedia Appendix 2.

#### Clinical Outcomes

For the main clinical outcome of BNP, the mean (minimum to maximum, SD) baseline value was 701.4 pg/mL (10.0-3852.1, 757.5), which decreased to 540.3 pg/mL (10.0-3739.7, 725.2) when measured at 6 months. After log transforming the BNP values and adjusting for the control variables in the linear regression (Table 3), there was a statistically significant decrease in BNP values at 6 months when compared with baseline. With a log-transformed outcome variable, an intuitive interpretation can be obtained from exponentiating the coefficient of interest. Therefore, exponentiating 0.47 (the coefficient for the 6-month follow-up variable) results in 1.59, indicating a 59% reduction in BNP after adjusting for the effect of the key demographic and control variables.

Linear regressions for the other clinical outcomes of sodium, creatinine, and LVEF indicated no significant change between baseline and 6-month values when holding control variables constant (Multimedia Appendix 2). Similarly, no change was found in the predicted survival score.

**Table 3:** Linear regression showing the effect of 6 months in the *Medly* program on B-type natriuretic peptide when controlled for key demographic and clinical variables.

Variables	Log (B-type natriuretic peptide) regression^a^	
	Coefficient (SE)	*P* value
6-month follow-up	−0.47 (0.11)	<.001
Onboarded from ward	0.20 (0.14)	.16
Left ventricular ejection fraction <40%	0.47 (0.13)	<.001
New York Heart Association class	0.36 (0.04)	<.001
Age (years)	0.02 (0.004)	<.001
Female	−0.30 (0.13)	.03
New to outpatient clinic	−0.01 (0.12)	.91
Intercept	3.51 (0.30)	<.001

^a^Number of observations = 486, adjusted *R^2^* = 0.22, *F* statistic (df) = 20.47 (7,478), *P*<.001.

#### Quality of Life

The mean (SD) MLHFQ total, physical, and emotional scores decreased from 53.2 (26.3) to 43.4 (26.0), 22.9 (11.8) to 17.4 (11.9), and 12.0 (7.5) to 10.2 (7.6), respectively. After adjusting for the control variable in the linear regressions (Table 4), improvements in MLHFQ scores were statistically significant for all 3 subscales. Specifically, the 6-month MLHFQ total scores were 9.8 points lower than their scores at baseline (*P*<.001), representing a change that is well above the 5-point change considered to be clinically meaningful. The physical and emotional subscales saw a decrease of 5.4 points (*P*<.001) and 1.5 points (*P*=.04), respectively. The linear regression of EQ-5D-5L scores found no significant change in the generic health status (Multimedia Appendix 1).

**Table 4:** Linear regressions showing the effect of 6 months in the *Medly* program on heart failure–related quality of life when controlled for key demographic and clinical variables.

Variables	MLHFQ^a^—total regression^b^		MLHFQ—physical regression^c^		MLHFQ—emotional regression^d^	
	Coefficient (SE)	*P* value	Coefficient (SE)	*P* value	Coefficient (SE)	*P* value
6-month follow-up	−9.78 (2.54)	<.001	−5.44 (1.18)	<.001	−1.51 (0.74)	.04
Onboarded from ward	4.74 (3.21)	.14	1.59 (1.48)	.28	−1.00 (0.94)	.29
Left ventricular ejection fraction <40%	−6.77 (2.94)	.02	−3.23 (1.36)	.02	−1.19 (0.86)	.17
New York Heart Association class	7.13 (1.03)	<.001	3.37 (0.48)	<.001	1.38 (0.30)	<.001
Age (years)	−0.64 (0.10)	<.001	−0.17 (0.04)	<.001	−0.21 (0.03)	<.001
Female	−1.66 (3.00)	.58	0.50 (1.39)	.72	−0.29 (0.88)	.74
New to outpatient clinic	−4.47 (2.69)	.10	−2.78 (1.24)	.03	−0.30 (0.79)	.71
Intercept	78.34 (7.29)	<.001	27.26 (3.37)	<.001	22.08 (2.13)	<.001

^a^MLHFQ: Minnesota Living with Heart Failure Questionnaire.

^b^Number of observations = 354, adjusted *R^2^* = 0.22, *F* statistic (df) = 15.12 (7, 346), *P*<.001.

^c^Number of observations = 354, adjusted *R^2^* = 0.19, *F* statistic (df) = 12.74 (7, 346), *P*<.001.

^d^Number of observations = 354, adjusted *R^2^* = 0.16, *F* statistic (df)=10.74 (7, 346), *P*<.001.

#### Self-Care

After 6 months in the *Medly* program, the mean (SD) SCHFI scores for maintenance, management, and confidence increased from 70.9 (16.8) to 78.5 (13.9), 64.2 (21.9) to 72.5 (19.1), and 67.3 (20.4) to 69.7 (20.2), respectively. After adjusting for the control variables in the linear regressions (Table 5), a statistically significant 7.76-point improvement in SCHFI maintenance scores (*P*<.001) and 8.46-point improvement in SCHFI management scores (*P*=.01) were found. These are close to or above the 8-point difference considered to be minimally clinically important. Finally, although there was a 2.55-point increase in SCHFI confidence scores, this change was not statistically significant.

**Table 5:** Linear regressions showing the effect of 6 months in the *Medly* program on self-care maintenance, management, and confidence when controlled for key demographic and clinical variables.

Variables	SCHFI^a^—maintenance regression^b^		SCHFI—management regression^c^		SCHFI—confidence regression^d^		
	Coefficient (SE)	*P* value	Coefficient (SE)	*P* value		Coefficient (SE)	*P* value
6-month follow-up	7.76 (1.67)	<.001	8.46 (3.29)	.01		2.55 (2.15)	.23
Onboarded from ward	6.02 (2.13)	.005	3.18 (3.81)	.41		5.08 (2.73)	.06
Left ventricular ejection fraction <40%	−2.13 (1.94)	.27	0.129 (3.45)	.97		−2.99 (2.48)	.23
New York Heart Association class	−1.56 (0.68)	.02	2.23 (1.30)	.08		−2.57 (0.87)	.003
Age (years)	0.10 (0.06)	.10	0.03 (0.12)	.81		−0.09 (0.08)	.25
Female	−1.40 (1.97)	.48	−0.37 (3.47)	.92		−3.82 (2.51)	.13
New to outpatient clinic	2.00 (1.78)	.26	−1.62 (3.24)	.62		4.87 (2.27)	.03
Intercept	68.53 (4.80)	<.001	55.87 (8.93)	<.001		79.67 (6.14)	<.001

^a^SCHFI: Self-Care of Heart Failure Index.

^b^Number of observations = 353, adjusted *R^2^* = 0.09, *F* statistic (df) = 5.75 (7, 345), *P*<.001.

^c^Number of observations = 199, adjusted *R^2^* = 0.02, *F* statistic (df) = 1.519 (7, 191), *P*=.02.

^d^Number of observations = 350, adjusted *R^2^* = 0.06, *F* statistic (df) = 4.17 (7, 342), *P*<.001.

## Discussion

### Principal Findings

This paper presents the results from a pragmatic pretest-posttest study aimed at determining the 6-month impact of an HF TM program that has been implemented as part of the standard of care in an outpatient heart function clinic. We found a 50% reduction for the primary outcome of HF-related hospitalizations and a 24% reduction in the number of all-cause hospitalizations after controlling for the key demographic and clinical variables of age, sex, NYHA class, LVEF, location of enrollment, and newness to the outpatient clinic. No significant changes were found for the other health service utilization outcomes of length of stay, ED visits, and outpatient clinic visits. However, because the *Medly* program was intended to fill the gap between scheduled clinic visits rather than to replace existing elements of care, the number of outpatient clinic visits was not expected to decrease. Thus, the fact that closer remote monitoring did not contribute to an increase in outpatient visits can be interpreted as a positive finding, particularly because no increases in ED visits were observed.

This study also showed that enrollment in the *Medly* program was associated with a significant reduction in BNP levels after 6 months, which, when interpreted alongside the lack of significant changes in other HF biomarkers (eg, creatinine), signals an improvement in patients’ physical health status. Finally, this study found statistically and clinically significant improvements in overall, physical, and emotional HF-related QoL as well as in self-care maintenance and self-care management.

### Comparison With Prior Work

In 2012, an RCT evaluating an earlier version of the *Medly* system found overall improvements in QoL compared with a control group in addition to significant improvements in BNP and self-care in patients who have been followed in the outpatient heart function clinic for more than 6 months. However, that study was underpowered (50 patients in each control and intervention arm) to show an impact on health service utilization outcomes. Now, with a larger sample size and a sustained program more firmly established within a clinic’s existing services, our study has replicated the original positive findings of that RCT in addition to showing a significant reduction in the number of hospitalizations.

These results are also consistent with systematic reviews and meta-analyses of RCTs [5,39,40], with the latest (at the time of writing) by Zhu et al [13] concluding that HF TM significantly reduces the number of all-cause and cardiac hospitalizations. Importantly, meta-analyses also point to a decreased risk of mortality, an outcome that could not be evaluated, given the lack of a control group in our study.

Practice-based evidence provides insights into an intervention’s real-world effectiveness and can further our understanding of when, how, and under what conditions interventions should be delivered. Therefore, the results of this study are most useful for decision makers or TM program planners when interpreted alongside the contextual detail provided in this and previous publications about the *Medly* program [26,27] in addition to overarching recommendations about when and how to implement HF TM interventions. For instance, a recent consensus statement from the Heart Failure Society of America broadly concluded that HF TM has the most impact when (1) patients are most at risk (eg, recent hospitalization, prone to fluid overload, and struggles with medication adherence), (2) rates of TM system usage and adherence are high, and (3) clear actions can be taken in response to the TM data [20]. In many respects, the results from this study are consistent with this consensus statement considering the high rates of patient adherence to the *Medly* program [27] and the fact that actionable feedback is sent to patients and that clinicians are part of the patient’s immediate circle of care. In addition, the statistical significance of the clinical variables used as controls in our impact analysis (ie, NYHA class and whether patients were enrolled immediately following an inpatient stay) is coherent with the idea that HF TM is most beneficial for patients most at risk. In light of this, these results are particularly meaningful because they suggest that an outpatient HF TM program can demonstrate impact under real-world conditions even when the inclusion criteria are left broad and decisions about enrollment are made based on clinical judgment.

### Limitations

The pragmatic study design, including the fact that much of the study data were collected as part of the standard of care, has methodological limitations. First, there was no control group, meaning that even if the analyses controlled for key demographic and clinical variables, the outcomes may have been influenced by subject maturation. Second, health service utilization data were restricted to events occurring at the 5 urban hospitals and institutes that make up the UHN. Therefore, although it is unlikely that patients would voluntarily seek HF care outside of the UHN, any such visits could not be analyzed, representing a major limitation of this study. This also applies to clinical outcome data, which were restricted to laboratory tests conducted at the UHN. However, because the *Medly* program does not necessarily aim to reduce the number of scheduled outpatient visits (which typically occur every 6 months), the impact of this limitation on laboratory data is expected to be minimal. Third, because the administrative data analyzed were not collected for the purposes of this study, important context is missing that would enable the drawing of more definitive conclusions. For example, although we can conclude that enrollment in the *Medly* program did not increase the overall number of outpatient visits, we did not have data that would allow us to further analyze what this means in terms of changes in scheduled versus unscheduled outpatient visits. Fourth, not all patients enrolled in the program consented to complete the questionnaires, leaving the analysis of patient-reported outcomes subject to selection bias. Similarly, allowing clinicians to use their judgment in determining who might benefit from the program may also contribute to difficulties in generalizing the results. Fifth, although the skew toward the enrollment of men in the *Medly* program is consistent with lower proportions of women in heart function clinics and HF research [41,42], this sex- and possibly gender-based limitation is an important consideration for the design of future TM interventions and research. Finally, because there is no defined duration of follow-up in the *Medly* program, not all patients who were initially a part of the program were enrolled for the full 6 months. However, analyses followed the intention-to-treat principle to mitigate any potential bias of excluding patients who left the program early.

### Conclusions

This study presented the results of a pretest-posttest study to evaluate the impact of an HF TM program by comparing the change in outcome measures at 6-month follow-up with those at baseline. After controlling for key demographic and clinical variables, regression analyses found that enrollment in the TM program led to a 50% reduction in the number of HF-related hospitalizations, a 24% reduction in all-cause hospitalizations, and a 59% reduction in BNP values. In addition, enrollment in the TM program was associated with statistically and clinically significant improvements in HF-related QoL and self-care maintenance and management. This study suggests that a real-world HF TM program, which provides patients with self-care support and active clinical monitoring by their existing care team, can reduce health service utilization and improve clinical, QoL, and patient self-care outcomes.

## Appendix

Multimedia Appendix 1 Baseline demographic and clinical characteristics of participants.

Multimedia Appendix 2 Regression outputs for length of stay, emergency department visits, outpatient clinic visits, sodium, creatinine, left ventricular ejection fraction, Seattle Heart Failure Model, and 5-level EuroQol 5-dimensional questionnaire.

## Abbreviations

BNP: B-type natriuretic peptide
BYOD: bring your own device
ED: emergency department
EMR: electronic medical record
EQ-5D-5L: 5-level EuroQol 5-dimensional questionnaire
HF: heart failure
IRR: incidence rate ratio
LVEF: left ventricular ejection fraction
MLHFQ: Minnesota Living with Heart Failure Questionnaire
MRP: most responsible physician
NP: nurse practitioner
NYHA: New York Heart Association
PMCC: Peter Munk Cardiac Centre
QoL: quality of life
RCT: randomized controlled trial
RN: registered nurse
SCHFI: Self-Care of Heart Failure Index
SHFM: Seattle Heart Failure Model
TM: telemonitoring
UHN: University Health Network
