# Supplementary material for: Outcomes of a Heart Failure Telemonitoring Program Implemented as the Standard of Care in an Outpatient Heart Function Clinic: Pretest-Posttest Pragmatic Study
Source: J Med Internet Res. 2020 Feb 6;22(2):e16538. doi: 10.2196/16538 (PMC7055875; doi:10.2196/16538)
Supplement: Multimedia Appendix 1 [file jmir_v22i2e16538_app1.docx]

**Multimedia Appendix 1: Baseline demographic and clinical characteristics of participants.**

| Characteristic | | Values |
| --- | --- | --- |
| Age (years), mean (SD) | | 58.3 (15.5) |
| **Sex, n (%)** | | |
|  | Male | 245 (77.8) |
|  | Female | 70 (22.2) |
| **Ethnicity, n (%)** | | |
|  | White | 144 (68.2) |
|  | Black | 15 (7.1) |
|  | Asian | 26 (12.3) |
|  | Other | 26 (12.3) |
| **Rurality, n (%)** | | |
|  | Urban | 121 (57.6) |
|  | Suburban | 61 (29.0) |
|  | Rural | 28 (13.3) |
| **Place of birth, n (%)** | | |
|  | Canada | 108 (51.2) |
|  | Elsewhere | 103 (48.8) |
| **Highest education achieved, n (%)** | | |
|  | Less than high school | 13 (6.2) |
|  | High school | 39 (18.6) |
|  | Trade or technical training | 31 (14.8) |
|  | College/university | 127 (60.5) |
| **Income in Can $, n (%)** | | |
|  | <15,000 | 30 (14.3) |
|  | 15,000-49,999 | 70 (33.4) |
|  | 50,000-74,999 | 28 (13.3) |
|  | >75,000 | 45 (21.4) |
|  | Preferred not to answer | 37 (17.6) |
| **Work, n (%)** | | |
|  | Working full time | 44 (20.9) |
|  | Working part time | 21 (10.0) |
|  | Retired for health reasons | 58 (27.5) |
|  | Retired for nonhealth reasons | 46 (21.8) |
|  | Unemployed/homemaker | 19 (9) |
|  | Other | 23 (10.9) |
| **Supplementary health insurance, n (%)** | | |
|  | Yes | 132 (63.2) |
|  | No | 77 (36.9) |
| **New York Heart Association functional classification, n (%)** | | |
|  | 2 or less | 143 (47.1) |
|  | 2-3 | 67 (22.0) |
|  | ≥3 | 94 (30.9) |
| Left ventricular ejection fraction, mean (SD) | | 31.8 (13.4) |
| **Left ventricular ejection fraction (categorical), n (%)** | | |
|  | Reduced ejective fraction (<40%) | 233 (74.7) |
|  | Preserved ejective fraction (≥40%) | 79 (25.3) |
| **Have a smartphone, n (%)** | | |
|  | Yes | 150 (72.1) |
|  | No | 58 (27.9) |
| **Comfort with smartphone, n (%)** | | |
|  | Not comfortable | 7 (4.5) |
|  | Somewhat comfortable | 31 (19.7) |
|  | Comfortable | 55 (34.4) |
|  | Very comfortable | 65 (41.4) |
| **Equipment used by patients, n (%)** | | |
|  | Full *Medly* kit (Bluetooth-enabled data transfer) | 222 (70.7) |
|  | Patients used personal phone and provided with peripheral devices (manual entry) | 54 (17.2) |
|  | Patients used all personal equipment (manual entry) | 38 (12.1) |
| **Location of onboard** | | |
|  | Heart function clinic (outpatient) | 235 (74.6) |
|  | Ward (inpatient) | 80 (25.5) |
| **Duration followed at the outpatient heart function clinic** | | |
|  | More than 6 months (old patients) | 183 (58.1) |
|  | Less than 6 months (new patients) | 132 (41.9) |
