# Supplementary material for: Outcomes of a Heart Failure Telemonitoring Program Implemented as the Standard of Care in an Outpatient Heart Function Clinic: Pretest-Posttest Pragmatic Study
Source: J Med Internet Res. 2020 Feb 6;22(2):e16538. doi: 10.2196/16538 (PMC7055875; doi:10.2196/16538)
Supplement: Multimedia Appendix 2 [file jmir_v22i2e16538_app2.docx]

**Multimedia Appendix 2: Regression outputs for length of stay, emergency department visits, outpatient clinic visits, sodium, creatinine, left ventricular ejection fraction, Seattle Heart Failure Model, and EQ-5D-5L**

Negative binomial regressions showing the effect of 6 months in the *Medly* program on total heart failure-related and all-cause length of stay when controlled for key demographic and clinical variables.

|  | Heart failure-related length of stay negative binomial regression^a^) | | | All-cause length of stay negative binomial regression^b^ | | |
| --- | --- | --- | --- | --- | --- | --- |
| Variables | Coefficient (SE) | IRR^c^ (SE) | P value | Coefficient (SE) | IRR | *P* value |
| 6-month follow-up | -.26 (.26) | .77 (.20) | .33 | -.16 (.22) | .86 (.19) | .47 |
| Onboarded from ward | 1.27 (.32) | 3.55 (1.13) | <.001 | 1.34 (.26) | 3.80 (.99) | <.001 |
| Left ventricular ejection fraction <40% | .05 (.31) | 1.06 (.33) | .86 | -.13 (.26) | .88 (.23) | .61 |
| New York Heart Association class | .32 (.11) | 1.38 (.15) | .003 | .31 (.09) | 1.36 (.12) | .001 |
| Age (years) | -.005 (.009) | 1.00 (.009) | .56 | -.005 (.008) | 1.00 (.008) | .49 |
| Female | -.29 (.33) | .75 (.24) | .37 | -.23 (.27) | .79 (.21) | .39 |
| New to outpatient clinic | .50 (.28) | 1.63 (.46) | .08 | .41 (.23) | 1.50 (.35) | .08 |
| Intercept | .46 (.72) | N/A^d^ | .52 | .84 (.59) | N/A^d^ | .152 |

^a^Number of observations = 606.

^b^Number of observations = 604.

^c^IRR: incidence rate ratio.

^d^N/A: not applicable

Negative binomial regressions showing the effect of 6 months in the *Medly* program on the number of heart failure-related and all-cause emergency department visits when controlled for key demographic and clinical variables.

|  | Heart failure-related emergency department visits (negative binomial regression^a^) | | | All-cause emergency department visits (negative binomial regression^b^) | | |
| --- | --- | --- | --- | --- | --- | --- |
| Variables | Coefficient (SE) | IRR^c^ (SE) | P value | Coefficient (SE) | IRR (SE) | *P* value |
| 6-month follow-up | -.69 (.51) | .50 (.26) | .18 | .27 (.27) | 1.31 (.35) | .31 |
| Onboarded from ward | -.69 (.79) | .50 (.40) | .39 | -.25 (.34) | .78 (.27) | .47 |
| Left ventricular ejection fraction <40% | .72 (.65) | 2.06 (1.35) | .27 | .30 (.32) | 1.35 (.43) | .35 |
| New York Heart Association class | -.12 (.20) | .89 (.18) | .56 | .05 (.11) | 1.05 (.12) | .66 |
| Age (years) | .02 (.02) | 1.02 (.02) | .21 | .02 (.01) | 1.02 (.01) | .09 |
| Female | .49 (.53) | 1.62 (.87) | .36 | .11 (.32) | 1.12 (.36) | .73 |
| New to outpatient clinic | -.44 (.56) | .64 (.36) | .43 | -.25 (.28) | .77 (.22) | .38 |
| Intercept | -4.81 (1.48) | N/A^d^ | .001 | -3.24 (.78) | N/A^d^ | <.001 |

^a^Number of observations = 606.

^b^Number of observations = 604.

^c^IRR: incidence rate ratio.

^d^N/A: not applicable

Negative binomial regression showing the effect of 6 months in the *Medly* program on the number of visits to the outpatient heart function clinic when controlled for key demographic and clinical variables.

|  | Outpatient clinic visits (negative binomial regression^a^) | | |
| --- | --- | --- | --- |
| Variables | Coefficient (SE) | IRR^b^ (SE) | P value |
| 6-month follow-up | .001 (.09) | 1.00 (.09) | .99 |
| Onboarded from ward | -.12 (.08) | 0.89 (.07) | .16 |
| Left ventricular ejection fraction <40% | .22 (.08) | 1.25 (.10) | .006 |
| New York Heart Association class | .08 (.03) | 1.08 (.03) | .005 |
| Age (years) | .001 (.002) | 1.00 (.002) | .54 |
| Female | -.06 (.08) | 0.95 (.08) | .50 |
| New to outpatient clinic | -.57 (.11) | 0.57 (.06) | <.001 |
| 6-month follow-up * Followed by clinic < 6 months | .86 (.14) | 2.36 (.33) | <.001 |
| Intercept | .45 (.18) | N/A^c^ | .013 |

^a^Number of observations = 604.

^b^IRR: incidence rate ratio.

^c^N/A: not applicable

Linear regressions showing the effect of 6 months in the *Medly* program on sodium and creatinine values when controlled for key demographic and clinical variables.

|  | Sodium regression^a^ | | Log (creatinine) regression^b^ | |
| --- | --- | --- | --- | --- |
| Variables | Coefficient (SE) | *P* value | Coefficient (SE) | *P* value |
| 6-month follow-up | .11 (.74) | .67 | .05 (.03) | .08 |
| Onboarded from ward | -.26 (.27) | .44 | .12 (.04) | .004 |
| Left ventricular ejection fraction <40% | -.58 (.34) | .07 | .01 (.03) | .73 |
| New York Heart Association class | -.28 (.32) | .01 | .08 (.01) | <.001 |
| Age (years) | -.005 (.11) | .61 | .007 (.001) | <.001 |
| Female | .45 (.01) | .17 | -.15 (.04) | <.001 |
| New to outpatient clinic | .33 (.33) | .24 | -.04 (.03) | .19 |
| Intercept | 139.02 (.28) | <.001 | 4.11 (.09) | <.001 |

^a^Number of observations = 497, adjusted *R*^2^ = 0.01, *F* statistic (df) = 2.00 (7, 489), *P*<.05.

^b^Number of observations = 497, adjusted *R*^2^ = 0.22, *F* statistic (df) = 20.48 (7, 489), *P*<.001.

Linear regression showing the effect of 6 months in the *Medly* program on LVEF, and predicted survival.

|  | Left ventricular ejection fraction ^a^ | | Seattle Heart Failure Model score regression^b,c^ | |
| --- | --- | --- | --- | --- |
| Variables | Coefficient (SE) | *P* value | Coefficient (SE) | *P* value |
| 6-month follow-up | 1.22 (1.06) | .25 | -.04 (.08) | .64 |
| Onboarded from ward | .61 (1.28) | .63 | .07 (.09) | .47 |
| Left ventricular ejection fraction <40% | - |  | - |  |
| New York Heart Association class | .48 (.43) | .30 | - |  |
| Age (years) | .22 (.04) | <.001 | - |  |
| Female | 2.15 (1.29) | .10 | - |  |
| New to outpatient clinic | -2.36 (1.12) | .04 | -.07 (.08) | .37 |
| Intercept | 18.45 (2.52) | <.001 | .86 (.06) | <.001 |

^a^Number of observations = 570, adjusted *R*^2^ = 0.08, *F* statistic (df) = 9.63 (6, 563), *P*<.001.

^b^Number of observations = 630, adjusted *R*^2^ = -0.003, *F* statistic (df) = 0.43 (3, 626), *P*<.73.

^c^Note that age, sex, NYHA class, and LVEF were used to calculate the SHFM score, so they were not included as control variables

Linear regressions showing the effect of 6 months in the *Medly* program on generic health status when controlled for key demographic and clinical variables.

|  | EQ-5D-5L regression^a^ | |
| --- | --- | --- |
| Variables | Coefficient (SE) | *P* value |
| 6-month follow-up | .04 (.02) | .07 |
| Onboarded from ward | .03 (.03) | .23 |
| Left ventricular ejection fraction <40% | .04 (.02) | .09 |
| New York Heart Association class | -.07 (.01) | <.001 |
| Age (years) | .002 (.001) | .003 |
| Female | .01 (.02) | .59 |
| New to outpatient clinic | .07 (.02) | .003 |
| Intercept | 0.52 (.06) | <.001 |

^a^Number of observations = 348, adjusted *R*^2^ = 0.20, *F* statistic (df) = 13.66 (7, 340), *P*<.001.
